# Early puberty and risk for type 2 diabetes in men

**DOI:** 10.1007/s00125-020-05121-8

**Published:** 2020-03-23

**Authors:** Claes Ohlsson, Maria Bygdell, Maria Nethander, Jenny M. Kindblom

**Affiliations:** 1 Centre for Bone and Arthritis Research, Institute of Medicine, the Sahlgrenska Academy at University of Gothenburg, Klinfarmlab, Vita Stråket 11, Sahlgrenska University Hospital, S-413 45 Gothenburg, Sweden; 2 Department of Drug Treatment, Sahlgrenska University Hospital, Region Västra Götaland, Gothenburg, Sweden; 3 Bioinformatics Core Facility, the Sahlgrenska Academy at University of Gothenburg, Gothenburg, Sweden; 4 Pediatric Clinical Research Center, Sahlgrenska University Hospital, Region Västra Götaland, Gothenburg, Sweden

**Keywords:** Childhood BMI, Cohort, Endocrinology, Epidemiology, National registers, Paediatrics, Puberty, School health records, Type 2 diabetes

## Abstract

**Aims/hypothesis:**

The association between pubertal timing and type 2 diabetes, independent of prepubertal BMI, is not fully understood. The aim of the present study was to evaluate the association between pubertal timing and risk of adult type 2 diabetes, independent of prepubertal BMI, in Swedish men.

**Methods:**

We included 30,697 men who had data for BMI at age 8 and 20 years and age at Peak Height Velocity (PHV), an objective assessment of pubertal timing, available from the BMI Epidemiology Study Gothenburg (BEST Gothenburg), Sweden. Information on type 2 diabetes (*n* = 1851) was retrieved from the Swedish National Patient Register. HRs and 95% CIs were estimated by Cox regression analysis. We observed violations of the assumption of proportional hazards for the association between age at PHV and the risk of type 2 diabetes and therefore split the follow-up period at the median age of type 2 diabetes diagnosis (57.2 years of age) to define early (≤57.2 years) and late (>57.2 years) type 2 diabetes diagnosis.

**Results:**

Age at PHV was inversely associated with both early (HR 1.28 per year decrease in age at PHV, 95% CI 1.21, 1.36) and late (HR 1.13, 95% CI 1.06, 1.19) type 2 diabetes. After adjustment for childhood BMI, the associations between age at PHV and both early (HR 1.24, 95% CI 1.17, 1.31) and late (HR 1.11, 95% CI 1.05, 1.17) type 2 diabetes were similar. Moreover, early age at PHV predicted insulin treatment of type 2 diabetes (OR 1.25 per year decrease in age at PHV, 95% CI 1.17, 1.33). Assuming a higher risk among those with an age at PHV below the median, the population attributable factor indicates that 15% fewer of the diagnosed individuals would have developed type 2 diabetes had they not reached puberty early.

**Conclusions/interpretation:**

These findings indicate that early puberty may be a novel independent risk factor for type 2 diabetes.

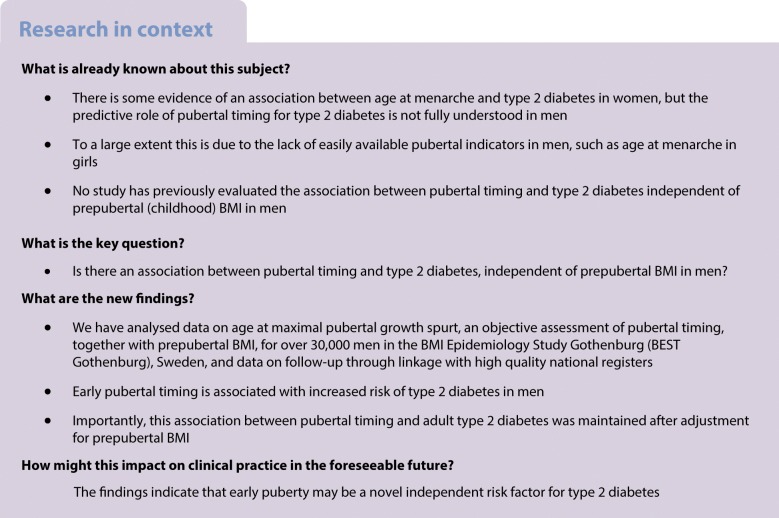



## Introduction

With the global rise in the prevalence of type 2 diabetes, identification of early risk factors that predispose individuals to type 2 diabetes is a priority. The strong association between a high adult BMI and risk of type 2 diabetes is well established, and prevention of adult obesity remains among the most important measures to reduce the burden of type 2 diabetes [1, 2]. We and others have recently demonstrated that being overweight during childhood and BMI change during puberty are associated with type 2 diabetes [3, 4]. These findings indicate that the increased risk of diabetes associated with elevated BMI may already be present in childhood and adolescence.

In addition to high BMI during childhood and adolescence, a few studies have indicated that early puberty is associated with increased risk of type 2 diabetes in women [5–9]. Information on age at menarche is often available and used as an estimate of pubertal timing in women, and recalled age at menarche has been shown to correlate rather well with actual age at menarche [10–12]. One study from the UK demonstrated that individuals with recalled age at menarche above the median had a lower risk of type 2 diabetes compared with those with recalled age at menarche below the median [8]. Recently, using the large amount of data available through the UK Biobank study, women with menarche before 11 years of age (20.2%) were found to have 76% higher risk of type 2 diabetes than those aged between 11 and 15 years at menarche [5]. Given the clear inverse association between childhood BMI and age at menarche in women [13–17], adjustment for the confounding effect of prepubertal BMI is important. Only one study, which analysed data on 1381 women in a British birth cohort born in 1946, had data available for prepubertal height and weight and was able to adjust for childhood BMI. In that study, the association between age at menarche and type 2 diabetes was strongly attenuated and no longer significant after adjustment for BMI at 7 years of age [9].

For boys, retrospective studies of pubertal timing are hampered by the lack of easily available pubertal indicators. In the UK Biobank study, recalled age at voice breaking is available as a pubertal marker [5]. The three self-reported categories of voice breaking in that study were ‘younger than average’, ‘about average’ and ‘later than average’, and the results showed higher risk for type 2 diabetes for the 4.3% who reported early voice breaking (OR 1.44, 95% CI 1.3, 1.59) and lower risk for the 5.9% who reported late voice breaking (OR 0.69, 95% CI 0.61, 0.77) compared with the approximately 90% that reported age at voice breaking ‘about average’ [5]. Age at voice breaking when reported by recall in adult life has not been validated [18]. Importantly, the analyses in the UK Biobank study could not be adjusted for prepubertal BMI, which is known to influence pubertal timing in men [19]. Therefore, the association between pubertal timing and type 2 diabetes, independent of prepubertal BMI, is unknown in men [5].

In the ongoing BMI Epidemiology Study Gothenburg (BEST Gothenburg), Sweden, both age at pubertal timing and information on childhood BMI and young adult BMI are available for a cohort of men born between 1945 and 1961. We used a modified Infancy-Childhood-Puberty model [20] to calculate age at peak height velocity (PHV), an objective assessment of pubertal timing, in men. Using these data, we have recently demonstrated an inverse association between prepubertal BMI at age 8 and age at PHV [19], and we also observed a clear secular trend towards earlier age at PHV in males from the 1940s up to the present [21].

In the present cohort study, we hypothesised that early age at PHV, independent of prepubertal BMI, is associated with increased risk of type 2 diabetes in men. The aim of the present study was to evaluate the association between pubertal timing and risk of adult type 2 diabetes, independent of childhood BMI, in Swedish men.

## Methods

### Study design

**Fig. 1:**
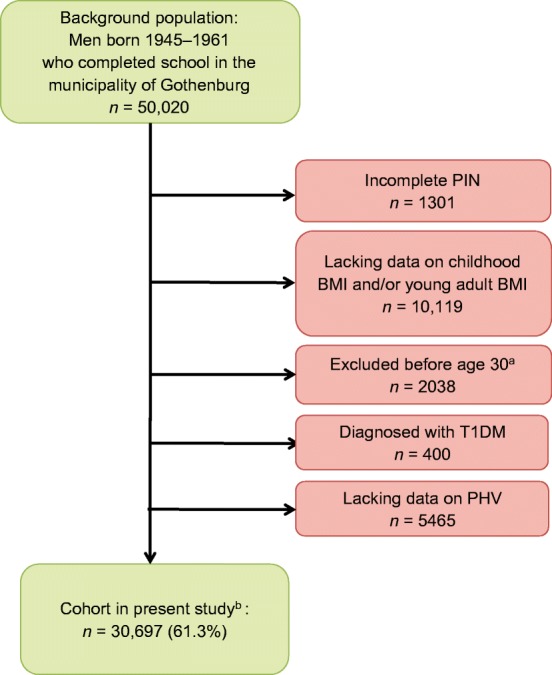
Flow chart of included individuals. ^a^Excluded due to any diabetes diagnosis, emigration or death; ^b^Followed until censoring due to a type 2 diabetes diagnosis, death, migration or until 31 December 2016. T1DM, type 1 diabetes

BEST Gothenburg was initiated with the overall aim of studying the impact of childhood and adolescent BMI and pubertal timing on adult diseases. We collected data on birthweight as well as directly measured height and weight from centrally archived school healthcare (SHC) records for all men born 1945 to 1961 in Gothenburg, Sweden, as previously described [22]. We also collected data on height and weight at young adult age (17.5–22.0 years of age) from military conscription tests. Conscription was mandatory until 2010 for all Swedish men. The study cohort was linked to high-quality national disease registers held at the Swedish National Board of Health and Welfare using the personal identity numbers (PINs) for the included subjects. Eligible individuals were those with an SHC record in the central archive and a 10-digit PIN (Fig. 1). Subjects with data available for calculation of both childhood BMI, young adult BMI and age at PHV were included in the present study (*n* = 30,697; Fig. 1 and Table 1). The following subjects were excluded before study start: (1) subjects with an incomplete PIN; (2) subjects lacking childhood BMI, young adult BMI or age at PHV; (3) subjects who died, emigrated, or were diagnosed with diabetes before 30 years of age; and (4) subjects who were diagnosed with type 1 diabetes at any time point (in total 38.7% excluded; Fig. 1). The 30,697 men included in the study were followed from 30 years of age until receiving a type 2 diabetes diagnosis (*n* = 1851) or censoring due to migration (*n* = 1750), death (*n* = 2841) or until 31 December 2016, whichever came first. Subjects who were censored during the study (i.e. those who emigrated or died) or were diagnosed with type 2 diabetes were included in the study until the date of censoring.

**Table 1:** Cohort description

Variable	Value
Exposures	
Childhood BMI (8 years; kg/m^2^)	15.7 ± 1.4
Young adult BMI (20 years; kg/m^2^)	21.4 ± 2.5
Mean (±SD) age at PHV (years)	14.1 ± 1.1
Median (IQR) age at PHV (years)	14.1 (13.4–14.8)
Birthweight (kg)^a^	3.6 ± 0.6
Childhood overweight	1920 (6.3)
Young adult overweight	2262 (7.4)
Country of birth	
Sweden	25,852 (84.2)
Other	4845 (15.8)
Education level^b^	
Elementary school	5425 (18.0)
Secondary education	13,194 (43.7)
Post-secondary education	11,561 (38.3)
Outcomes	
Type 2 diabetes	1851 (6.0)
Early (≤57.2 years)	926
Late (>57.2 years)	925
Insulin-treated type 2 diabetes	787
Mean (±SD) age at diagnosis (years)	56.6 ± 7.0
Median (IQR) age at diagnosis (years)	57.2 (52.5–61.9)

Data are presented as mean ± SD or *n* (%), unless stated otherwise; total cohort *n* = 30,697

Childhood overweight at 8 years of age was defined as BMI ≥17.9 kg/m^2^, while young adult overweight at 20 years of age was defined as BMI ≥25 kg/m^2^. The outcome type 2 diabetes includes new cases after 30 years of age

^a^Data on birthweight were available for *n* = 29,178

^b^Data on education level were available for *n* = 30,180

The ethics committee of the University of Gothenburg, Sweden, approved the study and waived the requirement for written or oral informed consent.

### Definition of study exposures

Prepubertal childhood BMI at 8 years of age and young adult BMI at 20 years of age were calculated using all paired height and weight measurements during the period between 6.5 and 9.5 years of age for prepubertal childhood BMI at 8 years of age, and in the period 17.5 to 22 years of age for young adult BMI at 20 years of age, as previously described [4]. All measurements within these intervals were used to construct a linear regression model and the data for individual subjects were then adjusted on the slope of the regression to obtain BMI at 8 and 20 years of age. After age adjustment, the BMIs were used to classify subjects as overweight or obese at 8 years of age (using the Centers for Disease Control and Prevention [CDC] cut-offs at 8 years of age of BMI ≥17.9 kg/m^2^ and BMI ≥20.0 kg/m^2^, respectively [23]) and at 20 years of age (based on the generally accepted cut-offs of BMI ≥25 kg/m^2^ and ≥ 30 kg/m^2^, respectively). Overweight refers to the population with a BMI above the overweight cut-off and includes both overweight and obese subjects at either 8 or 20 years of age. Details of birthweight were retrieved from SHC records.

To adequately calculate age at PHV in an unbiased manner, height measurements before, during and after the pubertal period are required. We calculated age at PHV according to the infancy–childhood–puberty (ICP) model [20] that we had further developed (using R [24]) as previously described [19, 25]. Age at PHV was defined as age at the maximum growth velocity during puberty and was estimated by the curve-fitting program.

The study subjects’ education level at age 45, categorised as low (=elementary school), middle (=secondary education) and high (=post-secondary education), and information on country of birth (categorised as Sweden if the subjects and both parents were born in Sweden, and other if the criteria were not fulfilled or information was missing) were retrieved from demographic registers at Statistics Sweden.

### Definition of study outcomes

Dates and diagnoses for the first appearance of a diagnosis of type 2 diabetes in any coding position were retrieved from the National Patient Register, initiated in 1964 and with full coverage in the Gothenburg region from 1972. A diagnosis of type 2 diabetes was defined according to the ICD system codes; E11 in ICD10 and 250 in ICD8 and ICD9 occurring for the first time after 30 years of age. The age cut-off of 30 years has been used by us and others [26] in order to avoid misclassifications between type 1 and type 2 diabetes, since these two entities are not separable in the diagnostic code systems ICD8 and ICD9 used until 1996 in Sweden. Information on insulin prescription was retrieved from the Prescribed Drug Register in Sweden, which has data available from 2005. Insulin treatment was defined as two or more prescriptions of insulin (Anatomical Therapeutic Chemical [ATC] classification code A10A) in the Prescribed Drug Register.

### Statistical analysis

We used the Student’s *t* test (unpaired) to compare means, Cox proportional hazards regression to analyse the association between exposures and events and a logistic regression model to analyse the association between exposures and insulin-treated type 2 diabetes. Non-linear associations were evaluated by inclusion of a quadratic term in the Cox proportional hazards regression model and possible interactions were assessed by addition of an interaction term in the Cox regression model. The interaction term included the two variables of interest multiplied by each other. Age at PHV did not show a significant interaction with childhood BMI. Kaplan–Meier survival plots were created with study subjects divided according to quartiles of age at PHV, and significance was tested using the logrank test between the groups. Descriptive data are presented as mean ± SD or *n* (%).

There were no missing values for the main variables. Birthweight and education level were available for a subsample of the entire cohort. Missing values were not imputed. Models including birthweight (*n* = 29,178) or education level (*n* = 30,180) only included the subgroup of boys with these variables available. Birthweight and BMI were log-transformed (using log_10_) when used in the Cox regression model.

The Population attributable factor (PAF) was estimated using the HR for age at PHV below vs above the median. The Cox regression was adjusted for birth year, country of birth and childhood BMI at 8 years of age. The PAF was calculated using the formula PAF = p_e_(1 − 1/HR), where p_e_ is the prevalence of exposure to pubertal timing below the median among cases of type 2 diabetes [27].

Kaplan–Meier survival and cumulative incidence plots and the test for proportionality were performed in R version 3.4.2 using the ‘survival’ and ‘rms’ packages [24, 28, 29] and all other analyses were performed in SPSS statistics (version 25, IBM, Armonk, NY, USA).

## Results

### Study cohort

In this study, 30,697 men born between 1945 and 1961 and who had available information on childhood BMI at age 8, young adult BMI at 20 years of age, and age at PHV were included and followed until 31 December 2016 (Fig. 1). Mean follow-up starting from 30 years of age was 30.7 years (941,673 person-years of follow-up). There were 1851 cases of type 2 diabetes before the end of follow-up and the median age at type 2 diabetes diagnosis was 57.2 years (Table 1).

### Association between age at PHV and type 2 diabetes

Age at PHV represents an objective assessment of pubertal timing. In the present cohort, mean age at PHV was 14.1 (SD 1.1) years. A Cox proportional hazards regression model adjusted for birth year and country of birth revealed an inverse linear association between age at PHV and the risk of type 2 diabetes (*p* = 1.3× 10^−19^, for non-linearity *p* = 0.41). When evaluating the association between age at PHV and the risk of type 2 diabetes, we observed violations of the assumption of proportional hazards, indicating that the strength of the association declined with follow-up time. We therefore split the follow-up period at the age of the median case of type 2 diabetes (57.2 years of age) and present the associations with early (≤57.2 years) and late (>57.2 years) type 2 diabetes separately in the subsequent analyses. Age at PHV was significantly associated with both early and late type 2 diabetes (Table 2). For every 1 year earlier age at PHV, the risk of early type 2 diabetes was 28% higher (HR per year decrease in age at PHV 1.28, 95% CI 1.21, 1.36) and the risk of late type 2 diabetes was 13% higher (HR 1.13, 95% CI 1.06, 1.19) (Table 2).

**Table 2:** Hazard ratios for early and late type 2 diabetes according to age at PHV in 30,697 Swedish men followed for a mean of 30.7 ± 7.5 years after age 30

Model	HR (95% CI) per year decrease	
	Early type 2 diabetes	Late type 2 diabetes
Base model	1.28 (1.21, 1.36)	1.13 (1.06, 1.19)
+ Adjustment for cBMI	1.24 (1.17, 1.31)	1.11 (1.05, 1.17)
+ Adjustment for aBMI	1.15 (1.08, 1.22)	1.05 (0.99, 1.11)
+ Adjustment for cBMI and aBMI	1.16 (1.09, 1.23)	1.05 (0.99, 1.11)

HRs (95% CI) per year decrease in age at PHV were calculated using Cox proportional hazards regression. Number of early (occurring ≤57.2 years) type 2 diabetes cases *n* = 926; total number in analysis *n* = 30,697. Number of late (occurring >57.2 years) type 2 diabetes cases *n* = 925, total number in analysis *n* = 24,513. All models are adjusted for birth year and country of birth with or without further adjustment for childhood BMI (at 8 years of age), young adult BMI (at 20 years of age), or both childhood BMI and young adult BMI

aBMI, young adult BMI; cBMI, childhood BMI

**Fig. 2:**
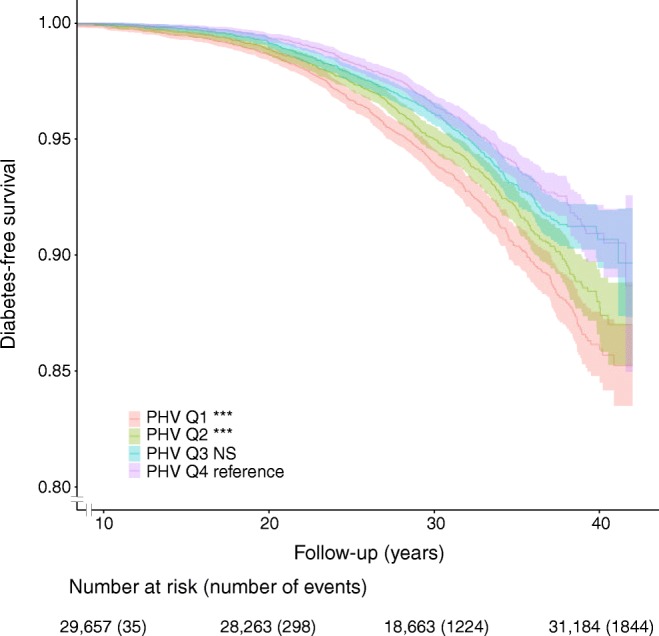
Kaplan–Meier curve of type 2 diabetes-free survival for age at PHV quartiles in 30,697 Swedish men followed for a mean of 30.7 ± 7.5 years after age 30. The graph shows type 2 diabetes-free survival according to pubertal timing (age at PHV) in quartiles. ***p< 0.001 for Q1 (earliest age at PHV quartile) and Q2 vs Q4 as assessed by logrank test. Q3 vs Q4 is non-significant

**Fig. 3:**
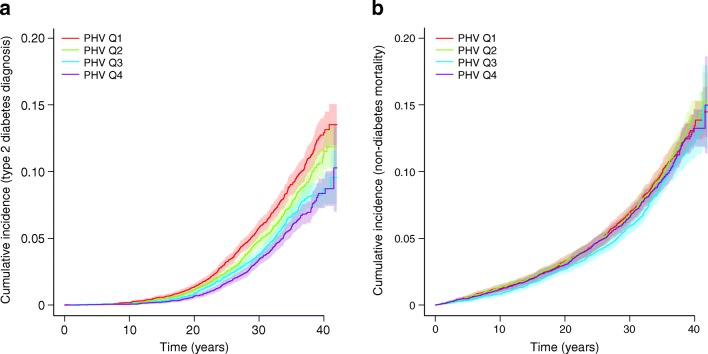
Cumulative incidence plots of type 2 diabetes (**a**) and type 2 diabetes-free mortality (**b**) according to age at PHV quartiles in 30,697 Swedish men followed for a mean of 30.7 ± 7.5 years after age 30. Type 2 diabetes (**a**) and type 2 diabetes-free mortality (**b**) are shown according to quartiles of age at PHV. Data are shown as proportions of 1 for events and shaded areas represent 95% CIs

Importantly, the risk of early type 2 diabetes was almost doubled for the subjects in the earliest age at PHV quartile, quartile 1 (Q1), compared with those in Q4 (HR 1.97, 95% CI 1.63, 2.38), and was clearly increased for subjects in Q2 and Q3 compared with those in Q4 (Table 3). In late type 2 diabetes, Q1 and Q2, but not Q3, had a moderately increased risk compared with Q4 (Table 4). A Kaplan–Meier survival plot confirmed a substantially increased risk of type 2 diabetes for the individuals with age at PHV in Q1 and Q2, but not for Q3, compared with individuals in Q4 (Fig. 2). Evaluation using cumulative incidence plots of type 2 diabetes and non-diabetes mortality did not indicate that there was any competing non-diabetes mortality disturbing the present finding of increased risk of type 2 diabetes for subjects in Q1, Q2 or Q3 compared with subjects in Q4 according to age at PHV quartile (Fig. 3).

**Table 3:** Hazard ratios for early type 2 diabetes according to quartiles of age at PHV in 30,697 Swedish men followed for a mean of 30.7 ± 7.5 years after age 30

Age at PHV quartiles	Range for age at PHV	Events	HR (95% CI)	
			Base model	Adjusted for cBMI
Q1 (*n* = 7674) vs Q4	9.3–13.4	317	1.97 (1.63, 2.38)	1.78 (1.47, 2.16)
Q2 (*n* = 7674) vs Q4	13.4–14.1	242	1.50 (1.23, 1.83)	1.46 (1.20, 1.79)
Q3 (*n* = 7675) vs Q4	14.1–14.8	205	1.26 (1.02, 1.54)	1.24 (1.01, 1.52)
Q4 (*n* = 7674)	14.8–17.9	162	Reference	Reference

HRs (95% CI) for type 2 diabetes for Q1–3 according to age at PHV with Q4 as reference were calculated using Cox proportional hazards regression. The base model is adjusted for birth year and country of birth, while the adjusted model also includes childhood BMI as a covariate. Cases of type 2 diabetes diagnosis *n* = 926, total number in analysis *N* = 30,697

cBMI, childhood BMI at age 8

**Table 4:** Hazard ratios for late type 2 diabetes according to quartiles of age at PHV in 24,513 Swedish men

Age at PHV quartiles	Range for age at PHV	Events	HR (95% CI)	
			Base model	Adjusted for cBMI
Q1 (*n* = 6128) vs Q4	9.3–13.4	278	1.33 (1.11, 1.59)	1.27 (1.05, 1.52)
Q2 (*n* = 6128) vs Q4	13.4–14.1	253	1.25 (1.04, 1.51)	1.22 (1.01, 1.47)
Q3 (*n* = 6129) vs Q4	14.1–14.8	193	0.97 (0.80, 1.18)	0.96 (0.79, 1.17)
Q4 (*n* = 6128)	14.8–17.9	201	Reference	Reference

HRs (95% CI) for late type 2 diabetes for Q2–Q4 according to age at PHV with Q1 as reference were calculated using Cox proportional hazards regression. The base model is adjusted for birth year and country of birth while the adjusted model also includes childhood BMI as a covariate. Cases of type 2 diabetes diagnosis *n* = 925, total number in analysis *N* = 24,513

cBMI, childhood BMI at age 8

Analyses according to age at PHV in quintiles with the median quintile as reference demonstrated a significant association between the early age at PHV quintile (quintile 1) and the risk of early (Table 5) but not late (Table 6) type 2 diabetes. Interestingly, individuals in the late age at PHV quintile (quintile 5) displayed a reduced risk of early type 2 diabetes (Table 5).

**Table 5:** Hazard ratios for early type 2 diabetes according to quintiles of age at PHV in 30,697 Swedish men followed for a mean of 30.7 ± 7.5 years after age 30

Age at PHV quintiles	Events	HR (95% CI)	
		Base model	Adjusted for cBMI
Quintile 1 (*n* = 6139)	277	1.59 (1.32, 1.92)	1.46 (1.20, 1.76)
Quintile 2 (*n* = 6140)	188	1.07 (0.87, 1.31)	1.04 (0.85, 1.28)
Quintile 3 (*n* = 6139)	176	Reference	Reference
Quintile 4 (*n* = 6140)	165	0.93 (0.76, 1.16)	0.95 (0.77, 1.18)
Quintile 5 (*n* = 6139)	120	0.69 (0.54, 0.87)	0.70 (0.56, 0.88)

HRs (95% CI) for type 2 diabetes for quintiles 1, 2, 4 and 5 according to age at PHV with quintile 3 as reference were calculated using Cox proportional hazards regression. The base model is adjusted for birth year and country of birth while the adjusted model also includes childhood BMI as a covariate. Cases of type 2 diabetes diagnosis *n* = 926, total number in analysis *N* = 30,697

cBMI, childhood BMI at age 8

**Table 6:** Hazard ratios for late type 2 diabetes according to quintiles of age at PHV in 24,513 Swedish men

Age at PHV quintiles	Events	HR (95% CI)	
		Base model	Adjusted for cBMI
Quintile 1 (*n* = 4902)	220	1.07 (0.88, 1.29)	1.03 (0.84, 1.25)
Quintile 2 (*n* = 4903)	205	1.04 (0.85, 1.26)	1.01 (0.83, 1.23)
Quintile 3 (*n* = 4903)	196	Reference	Reference
Quintile 4 (*n* = 4903)	159	0.82 (0.68, 1.01)	0.83 (0.67, 1.02)
Quintile 5 (*n* = 4902)	145	0.73 (0.59, 0.91)	0.75 (0.61, 0.93)

HRs (95% CI) for type 2 diabetes for quintiles 1, 2, 4 and 5 according to age at PHV with quintile 3 as reference were calculated using Cox proportional hazards regression. The base model is adjusted for birth year and country of birth while the adjusted model also includes childhood BMI as a covariate. Cases of type 2 diabetes diagnosis *n* = 925, total number in analysis *N* = 24,513

cBMI, childhood BMI at age 8

### Association between age at PHV and type 2 diabetes adjusted for childhood BMI and young adult BMI

We next evaluated whether the association between pubertal timing and risk of type 2 diabetes was independent of childhood BMI at 8 years of age (i.e. prepubertal BMI). Subjects with an age at PHV below the median had higher childhood BMI at 8 years of age (mean ± SD 15.9 ± 1.5 vs 15.5 ± 1.3 kg/m^2^, *p* = 6.3× 10^−17^) and young adult BMI at 20 years of age (21.9 ± 2.5 vs 20.9 ± 2.4 kg/m^2^, *p* = 6.1× 10^−10^) compared with subjects with age at PHV above the median. The associations between age at PHV and both early and late type 2 diabetes were similar after adjustment for childhood BMI (Tables 2, 3 and 4). When we adjusted the analyses for young adult BMI at 20 years of age (i.e. post-pubertal BMI; childhood BMI explains 36% of the variance in young adult BMI), the association between age at PHV and early type 2 diabetes was maintained, but the association with late type 2 diabetes was no longer significant (Table 2). When the model was adjusted for both childhood BMI and young adult BMI, there was a significant association between age at PHV and early, but not late, type 2 diabetes (Table 2). Thus, early age at PHV is associated with increased risk of both early and late type 2 diabetes independent of childhood BMI at 8 years of age.

Assuming a higher risk among those with an age at PHV below the median, the PAF of 15% indicates that 15% fewer of the individuals diagnosed with type 2 diabetes would have developed type 2 diabetes had they not had an early pubertal timing.

### The risk of insulin-treated type 2 diabetes

We then evaluated the association between age at PHV and the risk of a diagnosis of type 2 diabetes requiring insulin treatment (*n* = 787), i.e. type 2 diabetes with a worse global risk profile than type 2 diabetes without insulin treatment. We found that early age at PHV was associated with increased risk of insulin-treated type 2 diabetes (OR 1.25 per year decrease in age at PHV, 95% CI 1.17, 1.33). However, among subjects with type 2 diabetes (*n* = 1851), age at PHV did not significantly associate with insulin treatment (OR 1.06 per year decrease in age at PHV, 95% CI 0.97, 1.15).

### Adjustments for birthweight, education level and country of birth

Birthweight was directly associated with the risk of early (HR 0.81 per SD increase, 95% CI 0.77, 0.86) and late (HR 0.87 per SD increase, 95% CI 0.82, 0.93) type 2 diabetes. Adjustment for birthweight (*n* = 29,178) did not alter the association between age at PHV and risk of early (adjusted for birthweight: HR 1.26 per year decrease in age at PHV, 95% CI 1.19, 1.34; unadjusted: 1.27 per year decrease, 95% CI 1.19, 1.43) or late (adjusted for birthweight: HR 1.11 per year decrease, 95% CI 1.05, 1.18; unadjusted HR 1.11 per year decrease, 95% CI 1.05, 1.18) type 2 diabetes.

We next wanted to investigate whether the association between age at PHV and risk of type 2 diabetes could be partly explained by adult education level and therefore adjusted the Cox regression analysis for education level at age 45 categorised into low, middle and high groups. Education level itself showed a clear inverse association with the risk of early (low HR 2.34, 95% CI 1.95, 2.81 and middle HR 1.83, 95% CI 1.56, 2.15 vs high) and late (low HR 2.27, 95% CI 1.90, 2.71 and middle HR 1.61, 95% CI 1.37, 1.89 vs high) adult type 2 diabetes. Adjustment for education level did not alter the observed associations between age at PHV and early (HR 1.29 per year decrease, 95% CI 1.22, 1.36) or late (HR 1.12 per year decrease, 95% CI 1.06, 1.19) type 2 diabetes.

In the subpopulation of men born in Sweden with both parents born in Sweden (*n* = 25,852), similar associations as described for the complete cohort were seen for age at PHV with both early (HR 1.27 per year decrease, 95% CI 1.19, 1.35) and late (HR 1.14 per year decrease, 95% CI 1.07, 1.21) type 2 diabetes.

## Discussion

Childhood BMI is a known predictor of type 2 diabetes risk in men, but the predictive role of pubertal timing, independent of prepubertal BMI, for adult type 2 diabetes is not fully understood. We have collected information on height before, during and after puberty and used this data to calculate age at PHV, an objective assessment of pubertal timing, for 30,697 men in the BEST Gothenburg study. Using this data, we have demonstrated an inverse association between objectively assessed pubertal timing and the risk of type 2 diabetes in men. Importantly, we were able to adjust for childhood BMI at 8 years of age and show that the associations between pubertal timing and both early and late adult type 2 diabetes are independent of prepubertal BMI.

Male pubertal timing is not well studied. For girls, an inverse association between childhood BMI and age at menarche has been identified [17] and studies have established a secular trend of earlier menarcheal age since the mid-nineteenth century [30], partly explained by increased childhood BMI during the last 60 years [31]. Retrospective studies of male puberty are limited by the lack of easily available pubertal indicators, therefore, less is known regarding the determinants and the consequences of male pubertal timing. We have collected retrospective growth data, calculated age at PHV according to a modified infancy–childhood–puberty model [20] and used it as an objective assessment of pubertal timing in boys, as previously described [19, 21, 25]. Age at PHV is the age at the time of the maximum growth spurt, which occurs approximately 2 years after pubertal onset in boys [32]. Using age at PHV we recently reported a secular trend for pubertal timing from the 1940s onwards [21] and we have also demonstrated an inverse association between prepubertal childhood BMI and age at PHV [19]. In the current study we demonstrate that early age at pubertal timing is associated with increased risk of adult type 2 diabetes, independent of childhood BMI, in Swedish men.

The studies using age at menarche as assessment of pubertal timing suggest a risk pattern associated with early pubertal timing in women, including increased risk of type 2 diabetes and cardiovascular disease [5–9, 33, 34]. Among the studies in women we only found one that was able to adjust the association between early age at menarche and type 2 diabetes for prepubertal childhood BMI. The results indicated that the association was strongly attenuated by adjustment for prepubertal BMI and was no longer significant after the adjustment [9]. For men, the potential health consequences of early or late pubertal timing have not been evaluated in detail. In the UK Biobank study, self-reported age at voice breaking was inversely associated with the risk of type 2 diabetes. This finding is in line with the results using age at menarche in women in the same study [5], and with findings from previous studies demonstrating an association between early age at menarche and increased risk of type 2 diabetes [6–9]. The UK Biobank study looked at associations between extremes in self-reported pubertal timing (early-maturing [4.3%] and late-maturing [5.9%] boys) and risk of type 2 diabetes, and reported adverse health consequences for the 4.3% with self-reported early puberty [5]. In the present study, we demonstrate an inverse linear association between age at PHV, evaluated as a continuous variable, and the risk of type 2 diabetes. Importantly, as prepubertal BMI was not available in the UK Biobank study, the association between pubertal timing and type 2 diabetes in men could not be adjusted for childhood BMI [5] and, consequently, the association between pubertal timing and type 2 diabetes, independent of prepubertal BMI, is not known. As prepubertal BMI was available in the present study, we were able to demonstrate that age at PHV was associated with risk of type 2 diabetes independent of childhood BMI in men. This finding is further supported by the observation that age at pubertal timing was inversely associated with type 2 diabetes that required insulin treatment, i.e. type 2 diabetes with a worse global risk profile than those not requiring insulin treatment. Moreover, the notion that the significant inverse association between age at pubertal timing and risk of type 2 diabetes is also maintained after adjustment for education level and birthweight indicates that this association is robust to adjustment for available relevant confounders. Our analyses also demonstrated that late pubertal timing was associated with reduced risk of early type 2 diabetes, independent of prepubertal BMI.

The mechanisms behind the observed association between early pubertal timing and increased risk of type 2 diabetes in men are not clear. Since this is an observational study, we can only hypothesise on the possible factors mediating the observed association between early pubertal timing and increased risk of type 2 diabetes. The association between early menarche and increased risk of type 2 diabetes is reported to be completely [6, 8, 9] or partly [5, 7] attenuated by adjustment for adult age BMI, suggesting that increased adiposity might be an important mediator of the association in women. Some previous studies have shown a higher BMI and greater android fat mass at age 60–64 in men with earlier pubertal timing [18, 35], suggesting that pubertal timing may modulate BMI and adiposity during adult life in men. Unfortunately, BMI during middle age was not available in our study to test this hypothesis, but the notion that adjustment for young adult BMI at 20 years of age attenuated the association between pubertal timing and late type 2 diabetes aligns with the hypothesis that adult BMI could be partly involved in the mediation of this association. In a previous study, we demonstrated a higher amount of visceral adipose tissue in men with early puberty [25]. One may speculate that early puberty leads to the accumulation of visceral fat, and thereby increased cardiometabolic risk.

The limitations associated with the present study include the fact that information on BMI later in life was not available and that information on family history of diabetes and smoking was lacking. Another limitation is that the results may have limited generalisability to other ethnicities with a higher prevalence of type 2 diabetes, as Sweden and northwestern Europe have a low prevalence. Moreover, type 2 diabetes diagnoses were captured through hospital-based registers and it was not possible to verify a correct classification of the outcome diagnosis. However, the Prescribed Drugs Register captures all insulin dispensed at Swedish pharmacies and the strong association with insulin-treated type 2 diabetes supports a true association between pubertal timing and type 2 diabetes. The strengths of the study include that we used a well-powered population-based cohort with information on the objectively assessed pubertal timing and BMI available both before and after puberty, together with the long follow-up. Moreover, healthcare in Sweden is provided free of charge, which makes socioeconomic bias in diagnosis and representativeness unlikely.

In conclusion, we demonstrate that early pubertal timing is associated with increased risk of type 2 diabetes in Swedish men, independent of prepubertal BMI.

## Data Availability

Data cannot be shared publicly because of confidentiality under Swedish law. Registry data are available from the appropriate Swedish authorities (the Swedish National Board of Health and Welfare (https://www.socialstyrelsen.se/en), Statistics Sweden (https://www.scb.se/en), and the Swedish Defense Recruitment Agency. The BEST Gothenburg steering committee considers all collaboration requests (contact corresponding author).

## Abbreviations

BEST Gothenburg: BMI Epidemiology Study Gothenburg
PAF: Population attributable factor
PHV: Peak Height Velocity
PIN: Personal identity number
Q (in Q1, Q2, etc.): Quartile
SHC: School healthcare

## References

[1] Tuomilehto J, Lindstrom J, Eriksson JG (2001). Prevention of type 2 diabetes mellitus by changes in lifestyle among subjects with impaired glucose tolerance. N Engl J Med.

[2] Knowler WC, Barrett-Connor E, Fowler SE (2002). Reduction in the incidence of type 2 diabetes with lifestyle intervention or metformin. N Engl J Med.

[3] Bjerregaard LG, Jensen BW, Angquist L, Osler M, Sorensen TIA, Baker JL (2018). Change in overweight from childhood to early adulthood and risk of type 2 diabetes. N Engl J Med.

[4] Ohlsson C, Bygdell M, Nethander M, Rosengren A, Kindblom JM (2018). BMI change during puberty is an important determinant of adult type 2 diabetes risk in men. J Clin Endocrinol Metab.

[5] Day FR, Elks CE, Murray A, Ong KK, Perry JR (2015). Puberty timing associated with diabetes, cardiovascular disease and also diverse health outcomes in men and women: the UK Biobank study. Sci Rep.

[6] Dreyfus J, Jacobs DR, Mueller N (2015). Age at menarche and cardiometabolic risk in adulthood: the Coronary Artery Risk Development in Young Adults Study. J Pediatr.

[7] Elks CE, Ong KK, Scott RA (2013). Age at menarche and type 2 diabetes risk: the EPIC-InterAct study. Diabetes Care.

[8] Lakshman R, Forouhi N, Luben R (2008). Association between age at menarche and risk of diabetes in adults: results from the EPIC-Norfolk cohort study. Diabetologia.

[9] Pierce MB, Kuh D, Hardy R (2012). The role of BMI across the life course in the relationship between age at menarche and diabetes, in a British Birth Cohort. Diabet Med.

[10] Cairns BJ, Liu B, Clennell S (2011). Lifetime body size and reproductive factors: comparisons of data recorded prospectively with self reports in middle age. BMC Med Res Methodol.

[11] Cooper R, Blell M, Hardy R (2006). Validity of age at menarche self-reported in adulthood. J Epidemiol Community Health.

[12] Parent AS, Teilmann G, Juul A, Skakkebaek NE, Toppari J, Bourguignon JP (2003). The timing of normal puberty and the age limits of sexual precocity: variations around the world, secular trends, and changes after migration. Endocr Rev.

[13] Freedman DS, Khan LK, Serdula MK, Dietz WH, Srinivasan SR, Berenson GS (2002). Relation of age at menarche to race, time period, and anthropometric dimensions: the Bogalusa Heart Study. Pediatrics.

[14] Koprowski C, Ross RK, Mack WJ, Henderson BE, Bernstein L (1999). Diet, body size and menarche in a multiethnic cohort. Br J Cancer.

[15] Morris DH, Jones ME, Schoemaker MJ, Ashworth A, Swerdlow AJ (2010). Determinants of age at menarche in the UK: analyses from the Breakthrough Generations Study. Br J Cancer.

[16] Mumby HS, Elks CE, Li S (2011). Mendelian randomisation study of childhood BMI and early menarche. J Obes.

[17] Juul F, Chang VW, Brar P, Parekh N (2017). Birth weight, early life weight gain and age at menarche: a systematic review of longitudinal studies. Obes Rev.

[18] Ong KK, Bann D, Wills AK (2012). Timing of voice breaking in males associated with growth and weight gain across the life course. J Clin Endocrinol Metab.

[19] Bygdell M, Kindblom JM, Celind J, Nethander M, Ohlsson C (2018). Childhood BMI is inversely associated with pubertal timing in normal-weight but not overweight boys. Am J Clin Nutr.

[20] Karlberg J (1987). On the modelling of human growth. Stat Med.

[21] Ohlsson C, Bygdell M, Celind J et al (2019) Secular trends in pubertal growth acceleration in Swedish boys born from 1947 to 1996. JAMA Pediatr 173(9):e192315. 10.1001/jamapediatrics.2019.2315 10.1001/jamapediatrics.2019.2315 PMC6647355 31329245

[22] Ohlsson C, Bygdell M, Sonden A, Rosengren A, Kindblom JM (2016). Association between excessive BMI increase during puberty and risk of cardiovascular mortality in adult men: a population-based cohort study. Lancet Diabetes Endocrinol.

[23] Kuczmarski RJ, Ogden CL, Guo SS (2002). 2000 CDC growth charts for the United States: methods and development. Vital Health Stat.

[24] R Core Team (2017). R: a language and environment for statistical computing.

[25] Kindblom JM, Lorentzon M, Norjavaara E (2006). Pubertal timing is an independent predictor of central adiposity in young adult males: the Gothenburg osteoporosis and obesity determinants study. Diabetes.

[26] Zimmermann E, Bjerregaard LG, Gamborg M, Vaag AA, Sorensen TIA, Baker JL (2017). Childhood body mass index and development of type 2 diabetes throughout adult life–a large-scale Danish cohort study. Obesity (Silver Spring).

[27] Mansournia MA, Altman DG (2018). Population attributable fraction. BMJ.

[28] Harrell Jr FE (2016) rms: regression modelling strategies. R package rms_4.4–2.tar.gz. Available from https://cran.r-project.org/src/contrib/Archive/rms/. Accessed 21 Febr 2016

[29] Therneau T (2017) A package for survival analysis in S. version survival_2.41-3.tar.gz. Available from https://cran.r-project.org/src/contrib/Archive/survival/. Accessed 4 Apr 2017

[30] Wyshak G, Frisch RE (1982). Evidence for a secular trend in age of menarche. N Engl J Med.

[31] Kaplowitz PB (2008). Link between body fat and the timing of puberty. Pediatrics.

[32] Slora EJ, Bocian AB, Herman-Giddens ME (2009). Assessing inter-rater reliability (IRR) of Tanner staging and orchidometer use with boys: a study from PROS. J Pediatr Endocrinol Metab.

[33] Prentice P, Viner RM (2013). Pubertal timing and adult obesity and cardiometabolic risk in women and men: a systematic review and meta-analysis. Int J Obes.

[34] Widen E, Silventoinen K, Sovio U (2012). Pubertal timing and growth influences cardiometabolic risk factors in adult males and females. Diabetes Care.

[35] Sandhu J, Ben-Shlomo Y, Cole TJ, Holly J, Davey Smith G (2006). The impact of childhood body mass index on timing of puberty, adult stature and obesity: a follow-up study based on adolescent anthropometry recorded at Christ’s Hospital (1936-1964). Int J Obes.

